# PB2-Q591K Mutation Determines the Pathogenicity of Avian H9N2 Influenza Viruses for Mammalian Species

**DOI:** 10.1371/journal.pone.0162163

**Published:** 2016-09-29

**Authors:** Congrong Wang, Horace Hok Yeung Lee, Zi Feng Yang, Chris Ka Pun Mok, Zhi Zhang

**Affiliations:** 1 Department of Laboratory Medicine, Nanfang Hospital, Southern Medical University, GuangZhou, PR China; 2 Centre of Influenza Research, School of Public Health, HKU Li Ka Shing Faculty of Medicine, The University of Hong Kong, Hong Kong Special Administrative Region, PR China; 3 HKU-Pasteur Research Pole, School of Public Health, HKU Li Ka Shing Faculty of Medicine, The University of Hong Kong, Hong Kong Special Administrative Region, PR China; 4 State Key Laboratory of Respiratory Disease, National Clinical Research Center for Respiratory Disease, First Affiliated Hospital of Guangzhou Medical University, Guangzhou, PR China; 5 Department of Laboratory Medicine, Guangdong No.2 Provincial People’s Hospital, GuangZhou, PR China; Deutsches Primatenzentrum GmbH—Leibniz-Institut fur Primatenforschung, GERMANY

## Abstract

**Background:**

Influenza A subtype H9N2 is widespread and prevalent in poultry. It has repeatedly transmitted zoonotically to cause mild influenza-like illness in humans and is regarded as a potential pandemic candidate. In additon, the six internal genes of H7N9 and H10N8 viruses which caused infection in human in China as well as some of the highly pathogenic H5N1 strains are origined from H9N2. Previous studies have shown that the mammalian adaptation PB2-Q591K contributes to the pathogenicity of H5N1 and H7N9 viruses. However, the role of the PB2-Q591K mutation in H9N2 subtype is still not well understood.

**Methods:**

To define and compare the individual role of PB2-Q591K substitution in the PB2 gene segment of H9N2 in relation to polymerase activity, replication competence and the pathogenicity using in vitro and in vivo models.

**Results:**

The PB2-Q591K mutation in H9N2 virus enhanced the polymerase activity and virus replication in human NHBE cells when compared to the wild type strain. Mice infected with the PB2 mutant showed significant weight loss, higher virus replication and immune responses in the lungs.

**Conclusions:**

Our evidences suggest that the PB2-Q591K, in addition to the -E627K mutation in H9N2 enhanced the pathogenicity in mammalian host.

## Introduction

Influenza A subtype H9N2 viruses are the most widespread and prevalent influenza viruses in poultry found in Asia. Prior to the interventions from the government, the H9N2 virus isolation rates in Hong Kong’s live poultry markets was around 5% of all birds tested increasing to almost 20% in game-birds such as quail [1,2]. Since the poultries in Hong Kong are mainly imported from China, similar isolation rates are presumably common in the southern China region where market interventions applied in Hong Kong are not used. H9N2 viruses have repeatedly caused zoonotic human infections [3–5]. In most cases, it only causes a mild self-limited illness and thus the frequency of human infection is probably hugely under-estimated compared with H5N1 and H7N9 diseases which are usually much more severe to human.

The impact of H9N2 subtypes has been known to influence the human health through two pathways: On one hand, the 2+6 (2 surface genes + 6 internal genes) recombination was repeatedly found in the H5N1, H7N9 and H10N8 outbreaks in human being. In these outbreaks, the six internal genes of H9N2 viruses were found to combine with the HA and NA genes of other subtypes resulting to new virus strains and subsequently caused pathogenicity in human. On the other hand, mammalian adaptation in avian influenza viruses also raises the concern about its potential threat to human. Mutations in the PB2 gene of avian influenza virus have been known as important factors to the pathogenesis as PB2 is involved in the viral replication process and also functions in the determination of host range [6–7]. Studies of the highly pathogenic H5N1 or H7N9 viruses demonstrated that residue 627 of the PB2 plays a crucial role in adaptation and pathogenicity in mammalian hosts [6, 8]. For instance, infection of H5N1 with PB2-Q627K mutation in primary human lung epithelial cells and macrophages increases the virus replication as well as pro-inflammatory cytokines production and these factors have been shown to associate with the pathogenicity of H5N1 in human [9–11]. Recently, amino acid change at PB2-591 has been identified from the human pandemic H1N1, H5N1 and H7N9 viruses which is also related to the pathogenicity in mammalian hosts [8, 12–13]. Previous study has shown that mammalian adaptations at both 590S591R and 627K in pandemic H1N1 backbone have no synergistic effect when tested in combination [13]. Our previous study found that two amino acids residues located at the position 253 and 591 of the H9N2 PB2 gene were mutated after serial passages in mammalian cells while all other gene segments being unaffected [14]. The virus with both D253N and Q591K mutations in its PB2 showed higher cytokine induction and replication phenotype in our human cell models. In addition, infection of the mutant in balb/c mice caused significant weight loss when compared to the mice infected by the wild type strain. In this study, we extended our investigation to the role of the PB2-Q591K mutation when it is independently introduced in the genetic background of H9N2 virus.

## Materials and Methods

### Cells

Human embryonic kidney 293T and MDCK cells were maintained in Eagle’s minimal essential medium (MEM) containing 10% fetal calf serum and antibiotics. Normal Human Bronchial Epithelial (NHBE) Cells were obtained from Lonza and cultured according to the manufacturer’s protocol.

### Polymerase activity assay

Plasmids of the segments of the A/Quail/Hong Kong/G1/97 (H9N2/G1) and A/Duck/Hong Kong/Y280/97 (H9N2/Y280) viruses including those with single mutation at Q591K or E627K in the PB2 were generated. In brief, the RT-PCR products of viral RNA segments were cloned into the expression vector which contains dual promoters to produce viral RNAs and viral protein. The mutant PB2 constructs containing a mutation with amino acid at the position of interest were generated by QuikChange™ Site-Directed Mutagenesis Kit (Agilent Technologies). 293T cells monolayers were transfected with the luciferase reporter plasmid (pluci) and the internal control plasmid (phRL-CMV) together with the mix of PB2, PB1, PA and NP plasmids. After 24-hour incubation, cell extracts were prepared in 500 ul of lysis buffer. The luciferase levels were assayed with Luciferase Assay System (Promega) and detected by luminometer.

### Generation of recombinant viruses

All eight plasmids containing the full genome of the viruses were transfected into Human embryonic kidney (293T) cells to generate recombinant viruses according to the previous description [8].

### Cell Infection

Primary human bronchial epithelial cells were infected with the H9N2/G1 or H9N2/Y280 or their PB2 mutants at MOI of 0.01 according to the indicated condition. Evidences of viral replication from the supernantant of the infected cells were assessed by tissue infectious dose 50 in MDCK cells.

### Experimental mice infection

Specific pathogen free Female BALB/c mice (6–8 weeks old) were infected with 1.5x10^5^ PFU of each virus in 25 ul intranasally and were monitored daily for weight loss. Mice were sacrificed at the indicated days post-infection for virological and cytokine assays. The lungs were homogenized in PBS containing antibiotics. This study protocol was carried out in strict accordance with the recommendations and was approved by the Committee on the Use of Live Animals in Teaching and Research of the University of Hong Kong (CULATR 2270–10). Humane endpoints for animal’s experiments and methods were undertaken to minimize potential pain and distress. All animals were euthanized by Phentobarbital (200mg/kg, i.v.) at the end of the experiments or once they are severely sick which show more than one of the signs of the following (score >1, as one sign = 1): loss of weight more than 30%, respiratory signs, depression, diarrhea, cyanosis of the exposed skin, edema of the face and head, and neurological signs. No mice was fulfilled the above criteria and was euthanized before the end of the experiments.

### Quantitative analysis of cytokine levels

Expression levels of TNF-α, MIP-1α, MIP-1β, MCP-1, MCP-3, RANTES, KC and IP-10 in the lung homogenates and blood serum were quantitatively determined by flow cytometry–based immunoassay (Flowcytomix Multiplex, Bender MedSystems). In brief, both lung homogenates and blood serum was collected at indicated days of post-infection. 25ul of each sample was processed according to the manufacturer’s protocol. The amount of cytokines (pg/mL) in the samples was acquired on a BD LSRII (BD Bioscience) and was calculated by FlowCytomix Pro 2.3 software (Bender MedSystems).

### Virus titration

The amount of virus in the supernatant was titrated on MDCK cells and the titers were reported as tissue culture infectious dose units per 100ul (TCID_50_/100ul).

### Quantification of immune cells in the mice lungs

Whole lung cell suspensions from the mice were prepared in Dulbecco’s minimal essential media (DMEM) with 20% fetal calf serum following collagenase-DNase treatment and manual disruption. Red blood cells were removed by lysis buffer treatment. Lung cell suspensions were then incubated with anti-Fc block (anti- mouse CD16/CD32) to reduce non-specific antibody binding for 10 min prior to staining for 1 hr with fluorophore-conjugated antibodies specific for immune cell populations: CD11b-PE-Cy7, CD11c-PE, Ly6G/C-PerCP-Cy5.5, CD3-APC-eFluor 780, Dx5-Alexa Fluor 647. Cells were washed twice with PBS and fixed for 15 min with 2% paraformaldehyde and further stained for influenza NP-FITC antibody. Flow cytometry was performed on a FACSAria flow cytometer (BD Biosciences).

### Statistical analysis

Statistical significance of the difference between the experimental groups was determined by using unpaired, non-parametric Student’s t test. Values of p<0.05 were considered as significant.

## Results

### Q591K mutation in the PB2 gene of H9N2 virus enhances polymerase activity in mini-genome reporter assay

**Fig 1:**
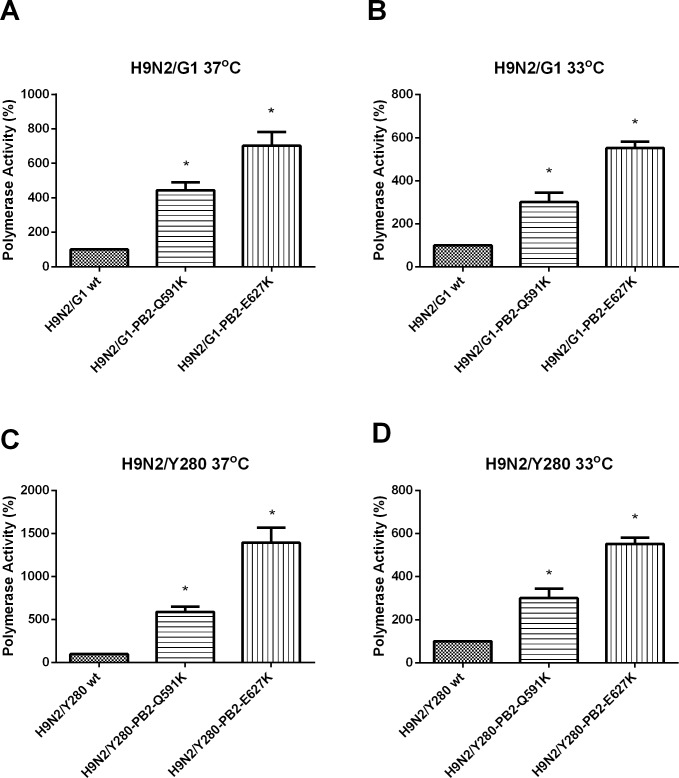
The polymerase activity of H9N2/G1 and H9N2/Y280 with PB2 mutations. 293T cells were transfected with plasmids containing H9N2/G1 or H9N2/Y280 PB2, PB1, PA, NP genes plus a control luciferase reporter plasmid and a viral UTR-driven luciferase reporter plasmid. After transfected cells were cultured at A) H9N2/G1-37°C and B) H9N2/G1-33°C C) H9N2/Y280-37°C D) H9N2/Y280-33°C for 24 hour, luciferase activity was then assayed in cell extracts. Results are the average of three experiments. The values were statistical analyzed by two tailed, paired t-test. *: p<0.05

Our previous study has shown that the amino-acid substitution Q591K and E627K in H9N2/G1 enhances polymerase activity in human 293T cells at 37°C [14]. We further examined and compared the effect of these mutations by mini-genome reporter assay using the backbones of the H9N2/G1 and the H9N2/Y280 at 33°C which represents the body temperature at the upper respiratory tract. Compared to the polymerase activity of the wild type H9N2/G1 (no mammalian signature in the PB2), both of the amino acid substitutions (PB2-Q591K and -E627K) resulted in a higher polymerase activity at 37°C and 33°C respectively in mammalian 293T cells (Fig 1A and 1B). However, the mutant PB2-E627K showed higher polymerase activity than the PB2-Q591K at both temperatures suggesting that PB2-Q591K mutation can only partially compensate the function of PB2-E627K. Similar results were observed using the H9N2/Y280 as the backbone under the same condition (Fig 1C and 1D).

### The PB2 mutants increase the virus replication in primary bronchial epithelial cells during the early time point after infection

**Fig 2:**
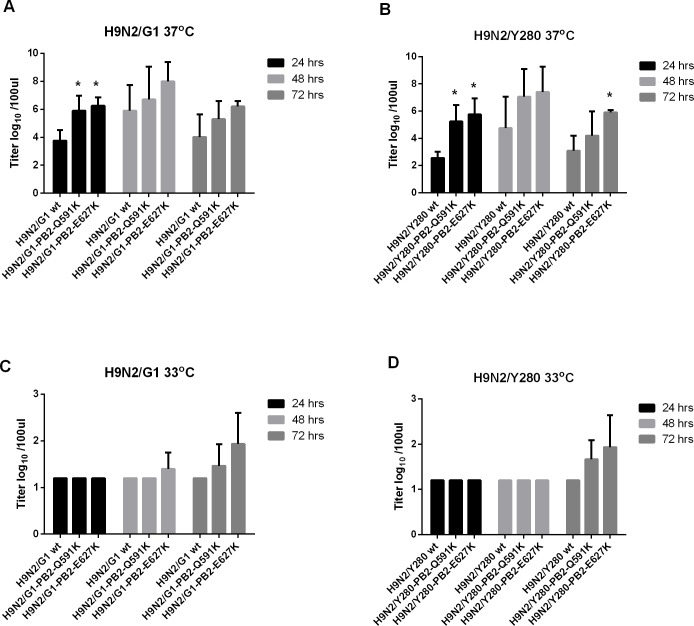
Virus replication of primary NHBE cells infected by H9N2 viruses. Primary NHBE cells were infected at MOI of 2 with H9N2/G1 or H9N2/Y280 and their PB2 variants. The virus titers were measured from the supernatant of 24, 48 and 72 hours post-infection by TCID50 assay. (A) H9N2/G1, 37oC (B) H9N2/Y280, 37oC (C) H9N2/G1, 33oC (D) H9N2/Y280, 33oC. The data is shown by the results of the mean value of three independent experiments. *: p<0.05

Initial infection and onward transmission of an influenza virus depends on efficient virus replication in the human respiratory tract. Normal human bronchial epithelial (NHBE) cells were infected at MOI of 0.01 at 33°C and 37°C with H9N2/G1 or H9N2/Y280 or their PB2 mutants. At 37°C, both PB2-Q591K and -E627K mutants from H9N2/Y280 and H9N2/G1 backgrounds showed significant higher virus replication than the wild type viruses at 24 hours post infection (Fig 2A and 2B). No difference of replication level was found between the wild type and the PB2 mutants at 48 and 72 hours post infection at the same temperature except the PB2-E627K mutant of H9N2/Y280, which showed significant higher replication than the wild type at 72 hours post infection. Both of the lineages grew ineffectively and no significant difference was found from the wild type strains and their PB2 mutants at 33°C (Fig 2C and 2D).

### The PB2-Q591K mammalian adaptation of H9N2 virus contributes to the pathogenicity in mice

**Fig 3:**
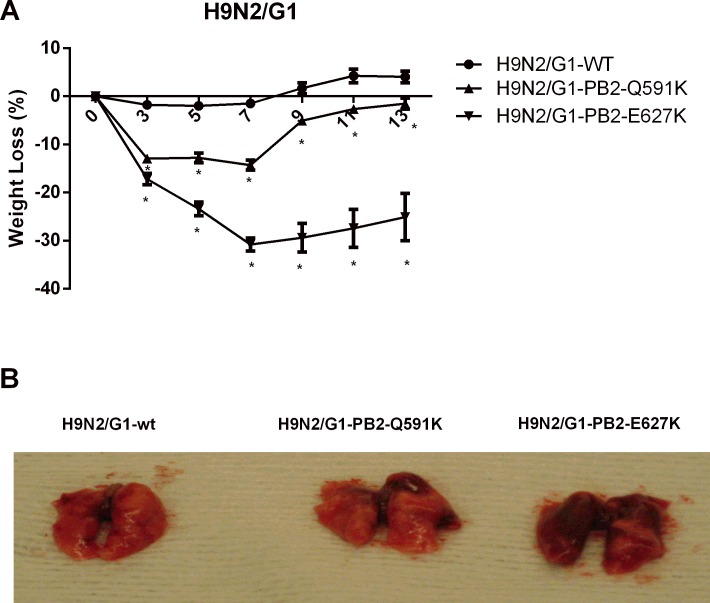
The weight change of the mice infected with the H9N2 and their PB2 variants. Female BALB/c mice were infected intranansally with 1.5x105 PFU of the H9N2/G1 and their PB2 mutants. Weight of the infected mice were expressed as percentage change compared to the weight at day 0. Results from each time point were expressed as mean ± SD of five mice. *:p<0.05. The data is the representative results from one of two independent experiments (B) The photographs from the lungs of the mice infected by the H9N2/G1 and their PB2 mutants.

**Fig 4:**
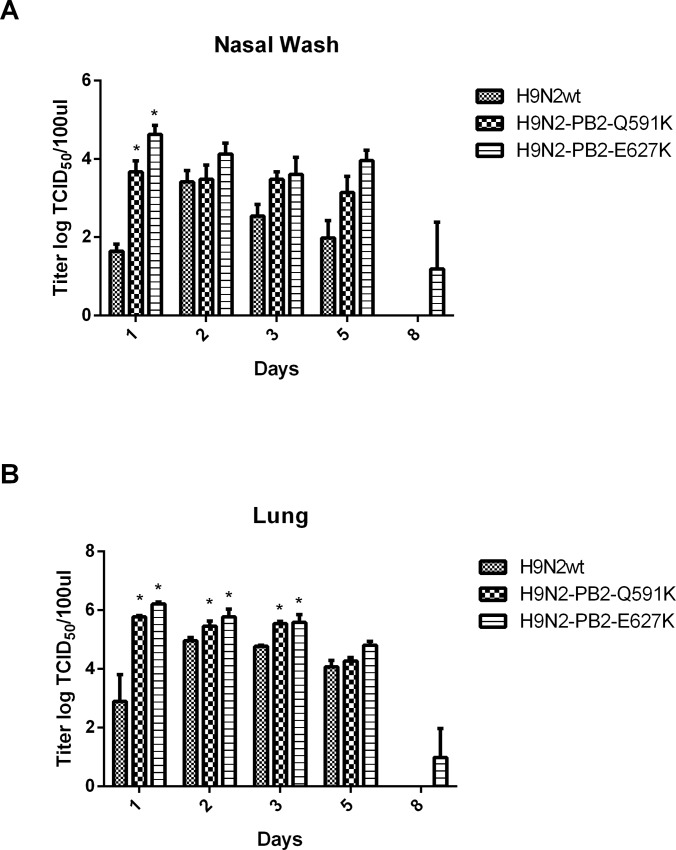
The virus titers from the nasal wash and the lung of the mice infected with the H9N2/G1 variants. Female BALB/c mice were infected intranasally with 1.5x105 PFU of the H9N2/G1 and their PB2 variants. (A) Nasal wash and (B) lung were harvested for virus titration at 3 and 6 days post-inoculation. Lungs were homogenized in 1 ml of PBS and 200 ul of PBS was used as nasal wash. The results from each group are represented by the average titer of six mice and are the representative results from one of two independent experiments. The virus titers were determined by TCID50 in MDCK cells. Results from each time point were expressed as means ± SD. *:p<0.05

To examine the role of H9N2 PB2-Q591K mutation to the pathogenicity in mice, we intranasally infected 6 to 8-week-old healthy female BALB/c mice (five mice in each group) with 1x10^5^ PFU of the recombinant wild type H9N2/G1 or mutants with PB2-Q591K or–E627K. Mice inoculated with the viruses were monitored for 14 days for weight loss and mortality (Fig 3A). No mice died after infection of any of the recombinant viruses even at the highest concentration we inoculated. The mice infected by the recombinant wild type H9N2/G1 virus showed less than 5% of weight loss in the next 14 days of post infection. The mice showed around 10–15% and 25–30% of weight loss at most after infection with the mutants of PB2-Q591K and -E627K respectively, suggesting that these two mutants contribute to the enhanced pathogenicity in mice. The appearance of the lungs isolated from the mice infected by wild type H9N2 or its PB2 mutants are shown in Fig 3B. Next, the virus replication in the respiratory tract and the cytokine induction from the mice infected by the recombinant wild type and their PB2 mutants were compared. No virus dissemination was found in the brain among all groups (data not shown). We determined the levels of virus replication in the nasal and lung of the virus-infected mice. Mice infected by the mammalian adapted H9N2 (PB2-Q591K and -E627K) showed higher replication level than the wild type in nasal wash samples collected at 3 days post infection (Fig 4A). A longer virus shedding (up to day 8) was observed in the group infected by the PB2-E627K mutant. The virus titer in the lung showed significant higher level in the groups of recombinant mutants compared to the wild type (Fig 4B).

**Fig 5:**
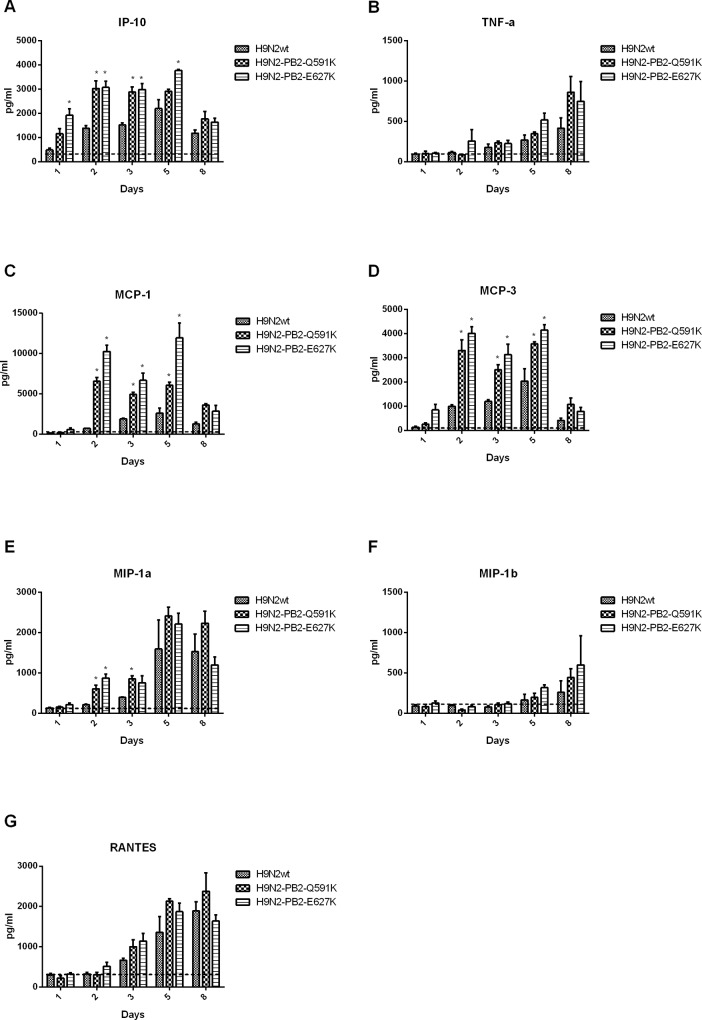
Cytokine expression in the lungs of H9N2/G1 virus infected mice. Cytokine (TNF-a, MIP1-a, MIP-1b, MCP-1, MCP-3, RNATES and IP-10) levels from infected lungs collected from days 3 and 6 post-inoculation were measured individually by the FlowCytomix system. Results from each time point are expressed as means ± SD of three infected mice (n = 3) and are the representative results from one of two independent experiments. Dot lines represent the level of mock. *:p<0.05

**Fig 6:**
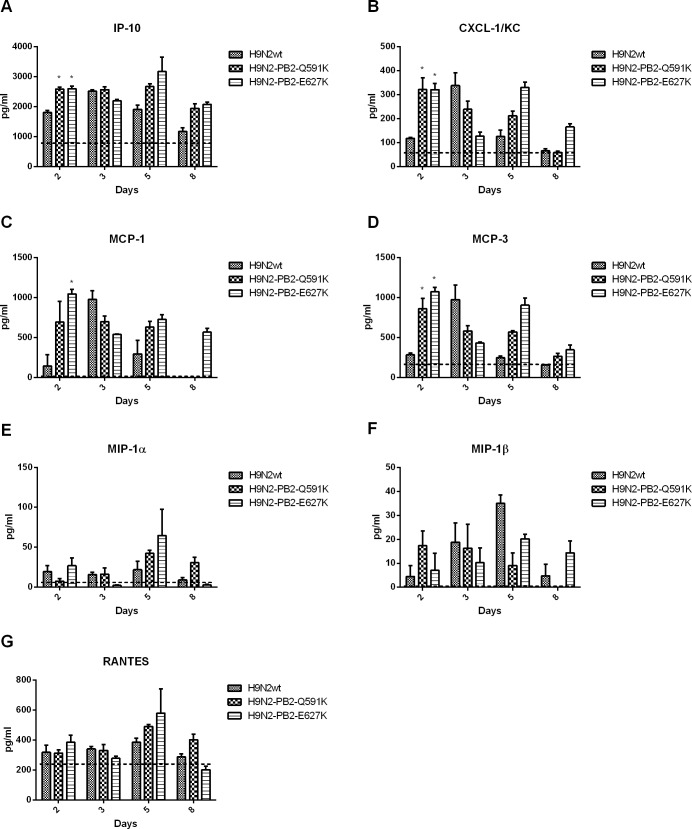
Cytokine responses in the serum of H9N2/G1 virus infected mice. Cytokine (IP-10, KC, MCP-1, MCP-3, MIP1-a, MIP-1b and RANTES) levels from the serum of infected mice were collected from days 3 and 6 post-inoculation and measured individually by the FlowCytomix system. Results from each time point are expressed as means of three infected mice (n = 3) and are the representative results from one of two independent experiments. Dot lines represent the level of mock. *:p<0.05

Clinical studies as well as in vivo models has been suggested that cytokine dysregulation contributes to the pathogenesis of H5N1 and H7N9 infection in humans. To have a better understanding to the role of H9N2 PB2-Q591K on cytokine induction, we compared the levels of several key proinflammatory cytokines in the lung and serum of the mice infected by wild type H9N2/G1 and PB2 mutants (Figs 5 and 6). In general, both the wild type and PB2 mutants triggered higher level of cytokines than the uninfected control. However, both PB2-Q591K and -E627K mutants induced significant higher amount of IP-10, MCP-3 and MCP-1 in the lung than the wild type virus, up to 5 days post infection. There was no difference on the levels of MIP-1α, MIP-1β, TNF-α and RANTES among all groups. In serum samples, both mutants triggered higher IP-10, KC and MCP-3 at day 2 of post infection but not found at the other days of infection.

**Fig 7:**
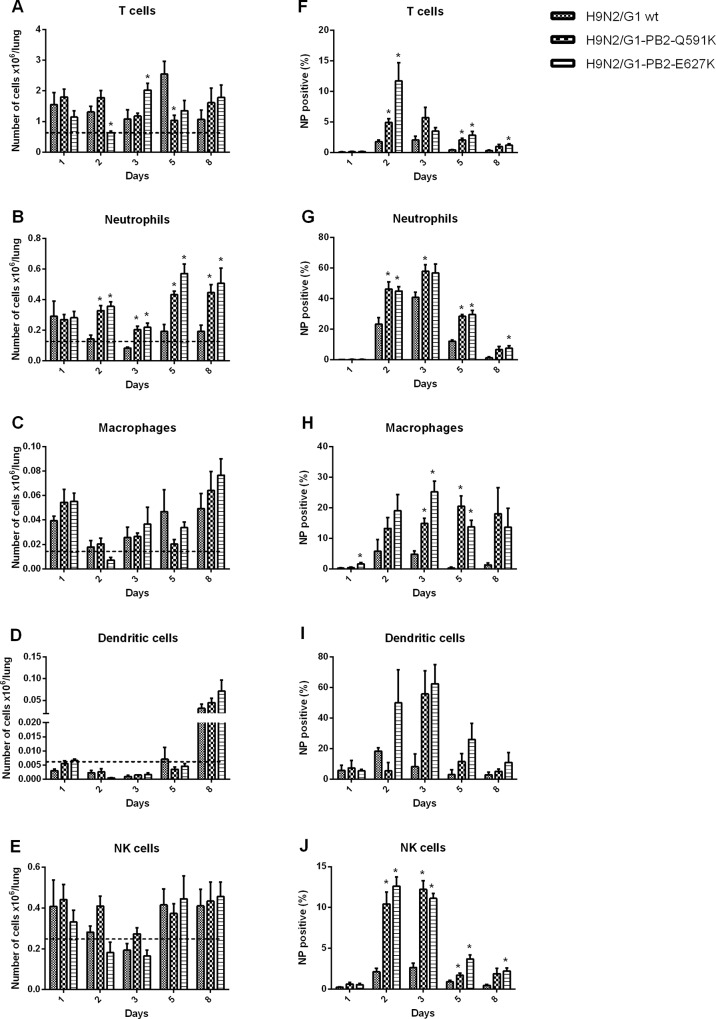
Lung cell characterization following infection with H9N2/G1 and the PB2 mutants. BALB/c mice were infected intranansally with 1.5x105 PFU of H9N2/G1 or the PB2 mutants. Cell sub-populations (A) T cells (B) Neutrophils (C) Macrophages (D) Dendritic cells (E) NK cells were collected from the lungs and were gated with antibodies at various times post-inoculation. The data shown represents the mean number of cells and standard deviations from four mice per time point, * p<0.05 compared to the H9N2/G1wt. (F-J) The percentage of NP positive cells from the subpoplutions. Gating: T cells(CD3+/CD11c-/CD11b-/Ly6G/C-), Neutrophils(CD11b+/CD11c-/CD3-/Ly6G/C+),Macrophages(CD11b+/CD11c-/CD3-/Ly6G/C-), Dendritic cells(CD11c+/CD11b-/Ly6G/C-/CD3-), NK cells(Dx-5+/CD3-). The data is the representative results from one of two independent experiments.

To further assess the immunity of the mice triggered by the PB2 mutants, we next determined the numbers of specific immune cell populations in the lungs using flow cytometry (Fig 7). Immune cells from the lungs of the mice infected by the H9N2/G1 wild type or the two PB2 mutants were identified by a combination of specific markers. Compared to the uninfected condition, mice infected with H9N2 viruses (Wild type or PB2 mutants) exhibited an increase in the numbers of macrophages (CD11b+, CD11c-, Ly6G/c-, CD3-), neutrophils (CD11b+, CD11c-, Ly6G/c+, CD3-), T cells (CD11b-, CD11c-, Ly6G/c-, CD3+) and NK cells (Dx5^+^, CD3^-^) since the first day of infection while the dendritic cells (CD11b-, CD11c+, Ly6G/c-, CD3-) increased at day 8. Among all the immune cells we tested, both of the PB2 mutants triggered higher influx of neutrophils than the wild type started from 2 days of post infection. Higher infection rates of T cells, neutrophils, macrophages and NK cells were found from both PB2 mutants but not in the wild type virus.

## Discussion

The H9N2 viruses have been repeatedly isolated from humans since 1999 [3,4,15]. While the viral determinants of pathogenicity in other avian influenza viruses such as H5N1 and H7N9 have been intensively studied, the role of these factors in H9N2 has not been fully investigated hitherto. We have previously reported that the PB2 mutations at the residues Q591K together with D253N are functionally relevant in H9N2 virus [14]. As PB2-Q591K mutation has been recently reported from human H5N1 and H7N9 isolates, we here aim to further investigate the pathogenic effects of this new identified PB2 mutation using in vitro and in vivo models with a parallel comparison to E627K, which is a well-studied mammalian adaptation. We showed that PB2-Q591K mutation in H9N2 backgrounds increases the polymerase activity as well as the early cycles of viral replication in human epithelial cells when compared to our wild type control which does not adapt to any mammalian signature. Recombinant H9N2 virus with the PB2-Q591K mutation clearly enhances the pathogenicity in mice as well as increasing virus replication in lung and triggering higher immune responses (proinflammatory cytokines and immune cells influx) compared with the wild-type control. However, consistent to our previous study using human H7N9 isolate as model, H9N2 virus with PB2-Q591K appeared to partially compensate for the lack of the PB2-E627K adaptation in polymerase activity and weight loss in mice [8]. In our in vivo experiments, although both mutants showed significant weight loss than the wild type virus, mice infected with PB2-Q591K mutant showed less weight loss and recovered faster than the one with PB2-E627K throughout the 14 days of post infection. However, both of them showed compatible level of replication in lung and no clear prolonged difference on immune responses suggesting that there is still an unknown factor to differentiate the pathogenicity between the two mutants.

Previous study from Mehle et al proposed that pandemic swine origin human influenza virus H1N1 that emerged in 2009 used the similar approach to overcome the species barrier from swine to humans [13]. A SR polymorphisms at position 590 and 591 of PB2 have been identified as substituting the function of the PB2-627K. However, no synergistic effect was observed when both of the G590S/Q591R and E627K mutations appear together suggesting that such mutations are a compensation substitution. Computational modeling and X-ray crystal structure of the “627” domain from the pandemic H1N1 PB2 protein showed that the PB2-590S591R can partially rescue the positive charge on the "627-domain” given that the PB2 retains 627E [12,13]. The introduction of these mutations into the PB2 of the pandemic H1N1 avian influenza increases higher polymerase activity but still, lower than the isogenic counterpart with PB2-E627K.

The host range of influenza viruses has shown to dependent on the compatibility between the virus components and the human host [16,17]. Several studies have demonstrated that the mammalian adaptation at PB2 switches the host factor dependence in avian and human host. It has been demonstrated that the polymerase activity and the replication of the influenza virus with PB2-E627K in mammalian hosts is positively regulated by importin-α1 and -α7 protein while -α3 generally plays a negative function to all influenza viruses [18, 19]. Moreover, virus with human-like PB2 627K but not avian-like PB2-627E displayed reduced pathogenicity and virus replication in importin-α7 knockout mice [20]. The mechanism on how the PB2 mutation affects the binding affinity with importin-α protein is still not known. While the PB2-Q591K mutation retains the similar structure and the charge on the surface of the same domain to the one with PB2-627K, it is interesting to further compare the interaction of the two PB2 with the importin-α protein given that PB2-Q591K showed a lower pathogenicity in our models.

Our results implicate that infection of H9N2 with specific PB2 mutation may possibly induce a more severe outcome in human. The cytokine storm and high virus replication were suggested as crucial factors to the pathogenicity of avian influenza virus in human [21–23]. We observed higher level of pro-inflammatory cytokines in the lung of the mice infected by the PB2-Q591K and E627K mutants and this phenomenon is likely due to the fact that more virus replication in the upper respiratory tract lead to more infection to immune cells resulting to more cytokines production. However, the lack of lethality in the mice infected by the mutants in high infectious dose suggested the mutations do not show a high virulence phenotype in the vivo model compared to H5N1 in which the unique property of hemagglutinin of the H5N1 is also shown as an important pathogenic factor.

In summary, our results showed the functional role of the PB2-Q591K mutation in H9N2 viruses. This adaptive change have been reported in human virus isolates of other avian viruses (e.g. H5N1 or H7N9 viruses) and was shown to increase the virulence, it is possibly that H9N2 with the similar mutation may increase the pathogenicity during human infection. Nevertheless, National Center for Immunization and Respiratory Diseases has included the PB2-Q591K mutation as one of the molecular determinants of important viral phenotypic characteristics in H5N1 virus (http://www.cdc.gov/flu/pdf/avianflu/h5n1-inventory.pdf). Taken together, our evidences suggested that this PB2 mutation in H9N2 enhanced the virus replication, immune responses and pathogenicity in mammalian host but did not contribute to the increasing of lethality.

## References

[1] Guan Y, Shortridge KF, Krauss S, Chin PS, Dyrting KC, et al H9N2 influenza viruses possessing H5N1-like internal genomes continue to circulate in poultry in southeastern China. (2000) J Virol. 74:9372–80. 10.1128/jvi.74.20.9372-9380.2000 11000205 PMC112365

[2] Kung NY, Guan Y, Perkins NR, Bissett L, Ellis T, et al The impact of a monthly rest day on avian influenza virus isolation rates in retail live poultry markets in Hong Kong. (2003) Avian Dis. 47:1037–41. 10.1637/0005-2086-47.s3.1037 14575106

[3] Peiris M, Yuen KY, Leung CW, Chan KH, Ip PL, et al Human infection with influenza H9N2. (1999) Lancet. 354:916–7. 10.1016/s0140-6736(99)03311-5 10489954

[4] Saito T, Lim W, Suzuki T, Suzuki Y, Kida H, et al Characterization of a human H9N2 influenza virus isolated in Hong Kong. (2001) Vaccine. 20:125–33. 10.1016/s0264-410x(01)00279-1 11567756

[5] Guo YJ, Wen LY, Wang M, Guo JF, Zhang Y, et al Characterization of internal genes of two strains of influenza A (H9N2) virus isolated from men. (2003) Zhonghua Shi Yan He Lin Chuang Bing Du Xue Za Zhi. 17:225–8. 15340563

[6] Hatta M, Gao P, Halfmann P, Kawaoka Y. Molecular basis for high virulence of Hong Kong H5N1 influenza A viruses. (2001) Science. 293:1840–2. 10.1126/science.1062882 11546875

[7] Neumann G and Kawaoka Y. Host range restriction and pathogenicity in the context of influenza pandemic. (2006) Emerg Infect Dis. 12:881–6. 10.3201/eid1206.051336 16707041 PMC3373033

[8] Mok CK, Lee HH, Lestra M, Nicholls JM, Chan MC, et al Amino acid substitutions in polymerase basic protein 2 gene contribute to the pathogenicity of the novel A/H7N9 influenza virus in mammalian hosts. (2014) J Virol. 88:3568–76 10.1128/JVI.02740-13 24403592 PMC3957932

[9] de Jong MD, Simmons CP, Thanh TT, Hien VM, Smith GJ, et al Fatal outcome of human influenza A (H5N1) is associated with high viral load and hypercytokinemia. (2006) Nat Med. 12:1203–7. 10.1038/nm1477 16964257 PMC4333202

[10] Hatta M, Hatta Y, Kim JH, Watanabe S, Shinya K, et al Growth of H5N1 influenza A viruses in the upper respiratory tracts of mice. (2007) PLoS Pathog. 3:1374–9. 10.1371/journal.ppat.0030133 17922570 PMC2000968

[11] Mok KP, Wong CH, Cheung CY, Chan MC, Lee SM, et al Viral genetic determinants of H5N1 influenza viruses that contribute to cytokine dysregulation. (2009) J Infect Dis. 200:1104–12. 10.1086/605606 19694514 PMC4028720

[12] Yamada S, Hatta M, Staker BL, Watanabe S, Imai M, et al Biological and structural characterization of a host-adapting amino acid in influenza virus. (2010) PLoS Pathog. 6:e1001034 10.1371/journal.ppat.1001034 20700447 PMC2916879

[13] Mehle A, Doudna JA. Adaptive strategies of the influenza virus polymerase for replication in humans. (2009) Proc Natl Acad Sci U S A. 106:21312–6. 10.1073/pnas.0911915106 19995968 PMC2789757

[14] Mok CK, Yen HL, Yu MY, Yuen KM, Sia SF, et al Amino acid residues 253 and 591 of the PB2 protein of avian influenza virus A H9N2 contribute to mammalian pathogenesis. (2011) J Virol. 85:9641–5 10.1128/JVI.00702-11 21734052 PMC3165745

[15] Guo YJ, Wen LY, Wang M, Guo JF, Zhang Y, et al Characterization of internal genes of two strains of influenza A (H9N2) virus isolated from men. (2003) Zhonghua Shi Yan He Lin Chuang Bing Du Xue Za Zhi. 17:225–8. 15340563

[16] Mehle A, Doudna JA. An inhibitory activity in human cells restricts the function of an avian-like influenza virus polymerase. (2008) Cell Host Microbe. 4:111–22. 10.1016/j.chom.2008.06.007 18692771 PMC2597520

[17] Moncorgé O, Mura M, Barclay WS. 2010 Evidence for avian and human host cell factors that affect the activity of influenza virus polymerase. J Virol. 84:9978–86. 10.1128/JVI.01134-10 20631125 PMC2937815

[18] Hudjetz B, Gabriel G. Human-like PB2 627K influenza virus polymerase activity is regulated by importin-α1 and -α7. (2012) PLoS Pathog. 8:e1002488 10.1371/journal.ppat.1002488 22275867 PMC3262014

[19] Gabriel G, Klingel K, Otte A, Thiele S, Hudjetz B. Differential use of importin-α isoforms governs cell tropism and host adaptation of influenza virus. (2011) Nat Commun. 2:156 10.1038/ncomms1158 21245837 PMC3105303

[20] Resa-Infante P, Thieme R, Ernst T, Arck PC, Ittrich H, et al Importin-α7 is required for enhanced influenza A virus replication in the alveolar epithelium and severe lung damage in mice. (2014) J Virol. 88:8166–79. 10.1128/JVI.00270-14 24829333 PMC4097772

[21] Cheung CY, Poon LL, Lau AS, Luk W, Lau YL, et al Induction of proinflammatory cytokines in human macrophages by influenza A (H5N1) viruses: a mechanism for the unusual severity of human disease? (2002).Lancet. 360:1831–7. 10.1016/s0140-6736(02)11772-7 12480361

[22] Chan RW, Yuen KM, Yu WC, Ho CC, Nicholls JM, et al Influenza H5N1 and H1N1 virus replication and innate immune responses in bronchial epithelial cells are influenced by the state of differentiation. (2010) PLoS One. 5:e8713 10.1371/journal.pone.0008713 20090947 PMC2806912

[23] Nicholls JM, Chan MC, Chan WY, Wong HK, Cheung CY, et al Tropism of avian influenza A (H5N1) in the upper and lower respiratory tract. (2007) Nat Med. 13:147–9. 10.1038/nm1529 17206149

